# Not All Fluctuations Are Created Equal: Spontaneous Variations in Thermodynamic Function

**DOI:** 10.3390/e26110894

**Published:** 2024-10-23

**Authors:** James P. Crutchfield, Cina Aghamohammadi

**Affiliations:** Complexity Sciences Center and Department of Physics, University of California at Davis, One Shields Avenue, Davis, CA 95616, USA; caghamohammadi@ucdavis.edu

**Keywords:** large deviation theory, thermodynamic formalism, fluctuation spectrum, entropy rate, fluctuation relations, nonequilibrium steady state, Maxwell’s Demon, information ratchet, Information Processing Second Law of Thermodynamics, 05.70.Ln, 89.70.-a, 05.20.-y, 05.45.-a

## Abstract

We identify macroscopic functioning arising during a thermodynamic system’s typical and atypical behaviors, thereby describing system operations over the entire set of fluctuations. We show how to use the information processing second law to determine functionality for atypical realizations and how to calculate the probability of distinct modalities occurring via the large-deviation rate function, extended to include highly correlated, memoryful environments and systems. Altogether, the results complete a theory of functional fluctuations for complex thermodynamic nanoscale systems operating over finite periods. In addition to constructing the distribution of functional modalities, one immediate consequence is a cautionary lesson: ascribing a single, unique functional modality to a thermodynamic system, especially one on the nanoscale, can be misleading, likely masking an array of simultaneous, parallel thermodynamic transformations that together may also be functional. In this way, functional fluctuation theory alters how we conceive of the operation of biological cellular processes, the goals of engineering design, and the robustness of evolutionary adaptation.

## 1. Introduction

Almost all processes—highly correlated, weakly correlated, or correlated not at all—exhibit statistical fluctuations. Often physical laws, such as the Second Law of Thermodynamics, address only typical realizations—those identified by Shannon’s asymptotic equipartition property [1] and that emerge in the thermodynamic limit of an infinite number of degrees of freedom and infinite time [2]. Indeed, our interpretations of the functioning of macroscopic thermodynamic cycles are so focused. What happens, though, during atypical behaviors, during fluctuations?

The limitation to typical behaviors is particularly a concern when it comes to information processing in thermodynamic systems or in biological processes, since fluctuations translate into errors in performing designed computing tasks or in completing the operations required for maintenance and survival, respectively. As a consequence, one realizes that the information processing second law (IPSL) only identifies thermodynamic functioning supported by a system’s typical realizations [3]. Now, since observing typical realizations is highly probable over long periods and goes to probability one in the thermodynamic limit, a definition of system functionality based on typicality is quite useful. However, this renders the IPSL substantially incomplete and practically inapplicable—ignoring fluctuations over finite periods and in microscopic systems. This is unfortunate. For example, while a system’s typical realizations may operate as an engine—converting thermal fluctuations to useful work—even “nearby” fluctuations (atypical, but probable realizations) behave differently, as Landauer erasers—converting the available stored energy to dissipate stored information. How do we account for functioning during fluctuations? And, over long periods, how, in fact, does a fluctuating system operate at all?

The following answers these questions by introducing constructive methods that identify thermodynamic functioning during any system fluctuation. It shows how to use the IPSL to determine functionality for atypical realizations and how to calculate the probability of distinct modalities occurring via the large-deviation rate function. The lesson is that, falling short of the thermodynamic limit, one cannot attribute a unique functional modality to a thermodynamic system.

To begin, the next section motivates our approach, reviewing its historical background and basic set-up. The development then reviews thermodynamic functioning in information engines and fluctuation theory proper, before bringing the two threads together to analyze functional fluctuations in a prototype information engine.

## 2. From Szilard to Functional Information Engines

Arguably, Szilard’s Engine [4] is the simplest thermodynamic device—a controller leverages knowledge of a single molecule’s position to extract work from a single thermal reservoir. As one of the few Maxwellian Demons [5] that can be completely analyzed [6], it exposes the balance between entropic costs dictated by the second law and thermodynamic functionality during the operation of an information-gathering physical system. The net work extracted exactly balances the entropic cost. As Szilard emphasized: while his single-molecule engine was not very functional, it was wholly consistent with the second law, only episodically extracting useful work from a thermal reservoir.

Presaging Shannon’s communication theory [7] by two decades, Szilard’s major contribution was to recognize the importance of the Demon’s information acquisition and storage in resolving Maxwell’s paradox [5]. The Demon’s informational manipulations had an irreducible entropic cost that balanced any gain in work. The role of information in physics [8] has been actively debated ever since, culminating in a recent spate of experimental tests of the physical limits of information processing [9,10,11,12,13,14,15] and the realization that the degree of the control system’s dynamical instability determines the rate of converting thermal energy to work [6].

Though many years ago, Maxwell [5] and then Szilard [4] were among the first to draw out the consequences of an “intelligent being” taking advantage of thermal fluctuations [16]. Szilard’s Engine, however, and ultimately Maxwell’s Demon are not very functional: Proper energy and entropy book-keeping during their operation shows their net operation is consistent with the second law. As much energy is dissipated by the Demon as it extracts from the heat bath [4]. There is no net thermodynamic benefit. Are there Demons that are functional?

Only rather recently was an exactly solvable Maxwellian engine proposed that exhibited functionality, extracting net work each cycle by decreasing physical entropy at the expense of positive change in a reservoir’s Shannon information [17]. There, the Demon generated directed rotation leveraging the statistical bias in a memoryless information reservoir to compensate for the transfer of high-entropy energy in a thermal reservoir to low-entropy energy that performed the rotational work. Since then, an extensive suite of studies analyzed more complex *information engines* [3,18,19,20,21,22,23,24,25,26,27,28]. Here, and in contrast with several of these studies, we emphasize engines that leverage information reservoirs with large, unrestricted memories while interacting with complex, correlated environments.

**Figure 1:**
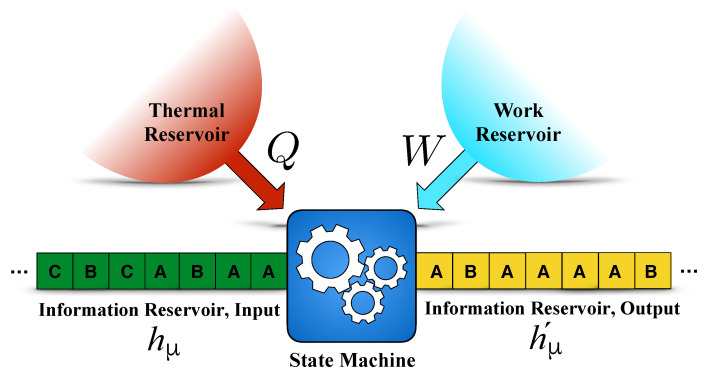
Information engine: A thermodynamically embedded state machine transforms symbols on the input tape with Shannon entropy rate $h_{\mu }$ to the output tape with Shannon entropy rate $h_{\mu }^{\prime }$. The input and output tapes comprise an information reservoir coupled, as are the thermal and work reservoirs, to the state machine. Tape symbols come from the same alphabet, e.g., as here, the set {A,B}. According to the information processing second law [3], by changing the Shannon entropies of the input and output symbol sequences, the information engine functions to convert heat *Q* to work *W* or work to heat depending on the sign of the entropy change $h_{\mu }^{\prime }-h_{\mu }$. Positive work and heat indicate energy flows into the Machine.

Figure 1 illustrates the general design for an information engine. The Demon, now denoted “State Machine”, is in contact with three reservoirs: thermal, work, and information. Each reservoir provides a distinct thermodynamic resource which the engine transforms. The thermal reservoir stores high-entropy energy; the work reservoir, low-entropy energy; and the information reservoir zero-energy Shannon information. The information reservoir consists of input and output tapes with cells storing discrete symbols.

The State Machine functions step by step. To process information on the tapes, it reads a symbol from an input cell and writes a symbol to an output tape cell and changes its internal state. The tapes then shift one cell presenting new input and output cells to the State Machine. In terms of the energetics, in the first step, a controller couples the symbol read from the input tape cell to the Machine. The controller may need positive or negative work from the work reservoir. The heat transfer is zero since, for our purposes here, we assume the process is relatively fast. In the second step, the state of the coupled cell–system transitions as a result of being in contact with the thermal reservoir. Then, the thermal reservoir induces a Markovian dynamics over the coupled cell–system joint states. This step is completely performed by the thermal reservoir and as a result there is heat transfer between the machine and thermal reservoir. The controller is absent and so the work carried out in this step is zero. In the third step, the controller decouples the output state from the machine state. Again, the work here can be nonzero, but the heat flow is zero.

There are three types of functioning. In the first, the state machine extracts heat from the thermal reservoir and performs work on the work reservoir by producing output symbol sequences with higher entropy than the input sequences. In this case, we say the machine functions as an *engine*. In the second, the machine decreases the output sequence entropy below that of the input by extracting work from the work reservoir and dumping that energy to the thermal reservoir. In this way, the machine acts as an *information eraser*. Finally, the third (non)functionality occurs when the machine uses (wastes) work energy to randomize output. Since the randomization of the input can happen spontaneously without wasting work—similar to the engine mode—we say the machine functions as a *dud*; it is a wasteful randomizer.

## 3. Environment and Engine Representations

There are two technical points that need to be called out here. First, we imagine the engine interacts with a complex environment. This means that we allow the input sequence to be highly correlated with a very long memory. Formally, the input sequence considered as a stochastic process is not necessarily Markovian. Denote the probability distribution over the input’s bi-infinite random variable chain by $P(\cdots X_{-1}X_{0}X_{1}\cdots )$, where $X_{t}$ is the random variable at time *t*. Then, the input sequence’s *Markov order R* is as follows:

$$\begin{array}{c} P\left(X_{t}|\cdots X_{-1}X_{0}X_{1}\cdots X_{t}\right)=P\left(X_{t}|X_{t-R}\cdots X_{t}\right)\;. \end{array}$$

And so, by complex environment we mean that input sequences to the machine have large *R*—the environment remembers long histories. Second, even though the machine has a finite number of states, we allow it to also have a long memory. This simply means that, via its states, the machine can remember the last, perhaps large, number of inputs.

One concludes from the first point about complex environments that Markov chains are not powerful enough to represent correlated inputs, especially for the general case we analyze. We need a less restrictive representation and so use *hidden Markov models* (HMMs), which are known to be more powerful in the sense that, using only a finite number of internal states, they can represent infinite Markov-order processes. We use HMMs to represent the mechanisms generating both input sequences and output sequences.

A process P’s HMM is given as a pair $\left\{𝓢,\{T^{\left(x\right)}:x\in A\}\right\}$. 𝓢 is HMM’s hidden states. $T^{\left(x\right)}$ for any particular *x* is a substochastic matrix or state-to-state transition matrix for transitions that generate symbol *x*. A is the alphabet of generated symbols.

Similarly, we conclude from the second point that more powerful machinery is needed to handle general stochastic mappings with a long memory. We use stochastic finite-state *transducers* [29] as they are powerful enough to represent the mappings we use in the following. (Several of the technical contributions stem directly from showing how to work directly with these powerful representations.)

A transducer representation is a pair $\left\{𝓢,\{T^{\left(x\to y\right)}:x\in A_{x},y\in A_{y}\}\right\}$. 𝓢 is the transducer’s states. $T^{\left(x\to y\right)}$ for any particular *x* and *y* is a substochastic matrix or state-to-state transition matrix for transitions that for input *x* generate symbol *y*. $A_{x}$ and $A_{y}$ are the alphabet for input and output symbols.

The following will demonstrate how these choices of representation greatly facilitate analyzing the dynamics and thermodynamics of information engines.

## 4. Thermodynamic Functioning: When Is an Engine a Refrigerator?

Thermodynamic functionality is defined in terms of the recently introduced *information processing second law* (IPSL) [3] which bounds the thermodynamic resources required, such as work, to perform a certain amount of information processing:

(1) $$\begin{array}{cc} \langle W\rangle & \leq k_{B}T\ln2\;\left(h_{\mu }^{\prime }-h_{\mu }\right)\;, \end{array}$$

where $k_{B}$ is Boltzmann’s constant and *T* is the environment’s temperature. The IPSL relates three macroscopic system measures: the input’s Shannon entropy rate $h_{\mu }$, the output’s entropy rate $h_{\mu }^{\prime }$, and the the average work 〈W〉 done on the work reservoir per engine cycle:

(2) $$\begin{array}{cc} h_{\mu } & =\lim_{l\to \infty }\frac{H[X_{0},X_{1},\cdots ,X_{l-1}]}{l}\;,\\ h_{\mu }^{\prime } & =\lim_{l\to \infty }\frac{H[X_{0}^{\prime },X_{1}^{\prime },\cdots ,X_{l-1}^{\prime }]}{l}\;,\;\mathrm{and}\\ \langle W\rangle & =\lim_{l\to \infty }\frac{1}{l}\sum_{w\in A^{l}}P\left(w\right)f\left(w\right)\;. \end{array}$$

**Figure 2:**
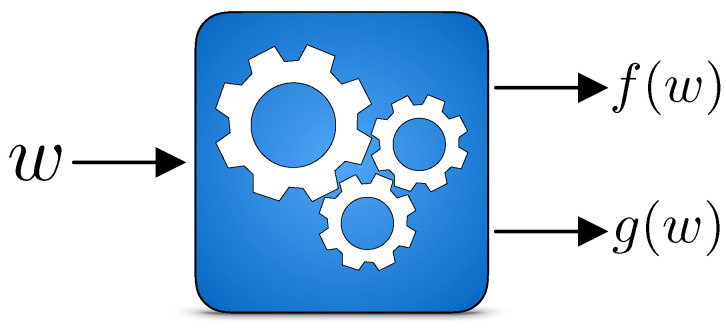
Input-dependent work and information: feeding in every single word *w*, on average the Machine generates work f(w) and information g(w).

Here, H[·] is the Shannon entropy of the specified random variables. f(w) is defined as follows. Since the machine stochastically maps inputs to outputs, a given input sequence *w* typically maps to many distinct output sequences. Then, f(w) denotes the average work carried out by feeding word *w* to the machine, averaging over all the possible mappings from *w*; see Figure 2.

That is, thermodynamic functioning is determined by the signs of 〈W〉 and $h_{\mu }^{\prime }-h_{\mu }$. Since there are two possible signs for each, there are four distinct cases. However, the IPSL forbids the cases 〈W〉>0 and $h_{\mu }^{\prime }-h_{\mu }<0$. And so, there are three thermodynamically functional modes: *engine*, *eraser*, and *ineffective randomizer*; see Table 1 [3]. When operating as an engine, the machine absorbs heat from the thermal reservoir and converts it to work by mapping the input sequence to a higher entropy-rate output sequence. Thus, the net effect is to randomize the input. When operating as an eraser, the machine reduces the input entropy by consuming work from the work reservoir and dumping it as high-entropy energy to the heat reservoir. In the third case, the machine does not function usefully at all. It is an ineffective randomizer, consuming work to randomize the input string. It wastes work, low-entropy energy.

## 5. A Functional Information Engine

**Figure 3:**
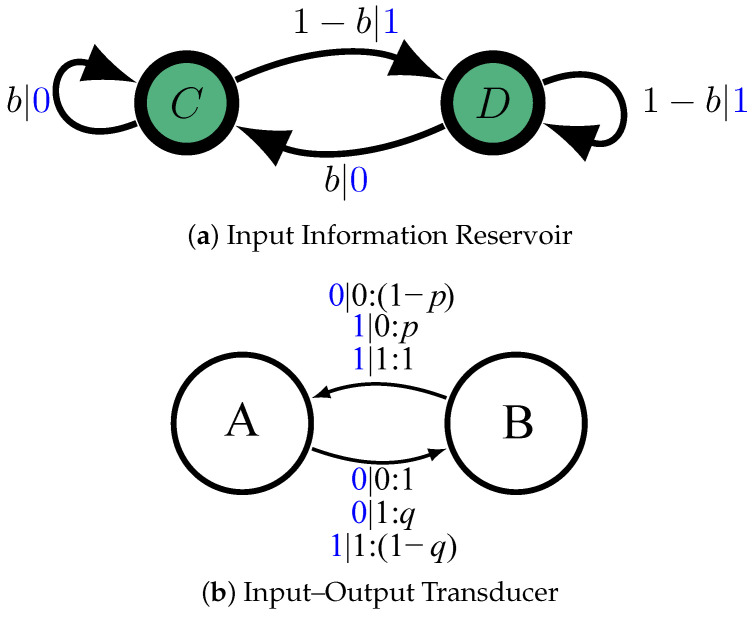
(**a**) Hidden Markov model that generates a biased coin input string $x_{t}x_{t+1}\ldots$ with bias Pr(X=0)=b. Edge labels x:p indicate a state-to-state transition of probability *p* that emits symbol *x*. (**b**) The information engine’s internal mechanism is a transducer. Its edge labels $x|x^{\prime }:p$ indicate a state-to-state transition of probability *p* taken on reading input symbol *x* that emits symbol $x^{\prime }$. (Reprinted from Ref. [3] with permission).

To ground these ideas, consider a prototype information engine—the *information ratchet* introduced in Ref. [3]. The engine, Figure 3, specifies the distribution of inputs and the states and transition structure of the engine’s state machine. The inputs come from flipping a coin with bias *b* for heads (“0”). That is, the input is a memoryless, independent, and identically distributed (IID) stochastic process. Its generating mechanism is depicted as the hidden Markov model in Figure 3a with two states, *A* and *B*. Together, the current state and transition taken determine the statistics of the emitted symbol. Similarly, the engine’s mechanism is represented by the finite-state transducer in Figure 3b. Transducer transitions are labeled. For example, if the machine is in state *B* and the input is 0, then with probability *p* the output emitted is 1 and the machine state changes to *A*. This is shown by an edge labeled by 1|0:p going from state *A* to *B*.

At this point, only the engine’s information processing has been specified. To design a physical system that implements the transducer, we first define the energetics for inputs and for machine states and transitions:

$$\begin{array}{cc} E(0) & =E(1)=0\;,\\ E(A) & =0\;,\;\mathrm{and}\\ E(B) & =e_{1}\;, \end{array}$$

where $e_{1}$ is a parameter. Second, we define the energetics for joint symbol-states:

$$\begin{array}{cccc} & E(A⊗0)=0\;,\; & & E\left(B⊗0\right)=-\epsilon_{1}\;,\\ & E\left(A⊗1\right)=-\epsilon_{2}\;,\; & & E\left(B⊗0\right)=+\epsilon_{3}\;. \end{array}$$

The energies $\epsilon_{i}$ are further constrained:

$$\begin{array}{c} e^{\left(\epsilon_{1}-\epsilon_{2}\right)/k_{B}T}=\frac{1-e^{-\left(\epsilon_{2}+\epsilon_{3}\right)/k_{B}T}}{1-e^{-\epsilon_{1}/k_{B}T}}\;. \end{array}$$

**Figure 4:**
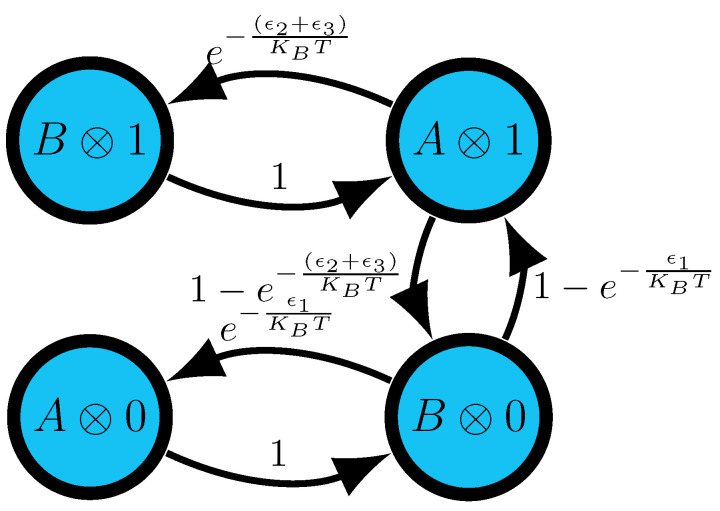
Markovian detailed balance dynamics induced by contact with the thermal reservoir in the coupled system (input symbol and machine state).

Third, we specify Markovian detailed-balanced dynamics over the coupled system (input + state machine) that is induced by the thermal reservoir; see Figure 4. To guarantee that this dynamic generates the same stochastic mapping as the transducer in Figure 3b, we must relate the energetics to stochastic-transition parameters *p* and *q*:

$$\begin{array}{cc} p & =1-e^{-\epsilon_{1}/k_{B}T}\\ q & =1-e^{-\left(\epsilon_{2}+\epsilon_{3}\right)/k_{B}T}\;. \end{array}$$

The average work carried out on the work reservoir is then as follows:

(3) $$\begin{array}{cc} \langle W\rangle \; & =\frac{k_{B}T}{2}[\left(pb-q+qb\right)\ln\left(q/p\right)\\ \; & \;\;+(1-b)q\ln(1-q)+pb\ln(1-p)]\;. \end{array}$$

See Ref. [3] for calculation details.

The Shannon entropy rates of input and output sequences can also be calculated directly:

(4) $$\begin{array}{cc} h_{\mu } & =H(b)\\ \; & \equiv -b\log_{2}b-\left(1-b\right)\log_{2}\left(1-b\right)\\ h_{\mu }^{\prime } & =\frac{H(b(1-p))}{2}+\frac{H((1-b)(1-q))}{2}\;. \end{array}$$

Thus, the energies $\epsilon_{1,2,3}$ and control *b* are the only free parameters. They control the engine’s behavior and, through the IPSL modalities in Table 1, its functionality. Reference [3] gives a complete analysis of this information engine’s thermodynamic functioning.

Summarizing for general information engines, one specifies the following:

1. Input process as an HMM;
2. Markovian detailed-balance dynamic over the coupled system of input and machine states as a finite-state transducer with consistent energy assignments.

This prepares us to analyze fluctuations in an information engine interacting with the complex environment specified by the input process.

## 6. Engines in Fluctuating Environments: The Strategy

Hidden in this and often unstated, but obvious once realized, Maxwellian Demons cannot operate unless there are statistical fluctuations. Szilard’s Engine cleverly uses and skirts this issue since it contains only a single molecule whose behaviors, by definition, are nothing but fluctuations—single realizations. There is no large ensemble over which to average. The information gleaned by the engine’s control system (Demon/Machine) is all about the “fluctuation” in the molecule’s position. And, that information allows the engine to *temporarily* extract energy from a heat reservoir. In short, fluctuations are deeply implicated in the functioning of thermodynamic systems. The following isolates the underlying statistical mechanisms.

The distinct types of thermodynamic functioning—engine, eraser, or dud—are based on three average quantities: average work produced 〈W〉, the input sequences’ Shannon entropy rate $h_{\mu }$, and the output sequences’ Shannon entropy rate $h_{\mu }^{\prime }$ [3,18,19,20,21,22,23,24,25,26,27,28]. As a result, their definitions concern the thermodynamic limit of infinitely long sequences being fed into the machine. Of course, the situation is practically quite different: the engine works with and operates due to finite-length sequences.

To overcome this—and so to develop a theory of functional fluctuations—the following is burdened with precisely delineating the limitations inherent in the infinite-length definitions above. It shows that, for any finite length, the functionality definitions are limited to describing properties of only a unique subset of events—the so-called typical set of realizations as identified by the asymptotic equipartition property of information theory [1]. To do this, first we redefine the three quantities—work and entropy rates—as averages over all the possible input sequences of a given length. Second, we define three new unweighted-average quantities, but this time they are explicitly limited to typical realizations. Third, we demonstrate that the differences between the first three averages and the second three can be made arbitrarily small. Since the second kind of averages are unweighted, the closeness result tells us that the average quantities are features of the typical set and not of any other subset of the input sequences. In point of fact, they do not describe atypical behaviors (statistical fluctuations) and so cannot be used to define thermodynamic functions arising from fluctuations.

One technical reason behind this result is that, for the three averages, the functions being averaged are linearly bounded from above by the input-sequence length. The conclusion is that the original quantities can give information only about system functionality for the specific subset of typical realizations. Of course, since observing realizations in this subset is highly probable for long sequences and has probability one in the thermodynamic limit of infinite length, the original functionality definition is quite useful. Our goal, though, is to show just how incomplete it is and in important ways that must be overcome to analyze fluctuations in functioning.

In short, the following consistently extends the original definitions to other realization subsets—the fluctuations or atypical sets. The net result is that the theory covers the set of any realization for any finite length. Given that, we introduce a method to calculate the new functionality for these different fluctuation subsets. This completes the picture of functional fluctuations for finite, but long, lengths. We go on to find the large deviation rate for the new definition of functionality. An important contribution in this is that all of the results also apply to input sequences and machines with long memories, given that the latter are stochastic finite-state machines. This should be contrasted with developments, cited above, that assume memoryless or order-1 Markov systems. We return to discuss related work at the end, once the results are presented.

## 7. Functioning Supported by Typical Realizations

A picture of a system’s behavioral fluctuations can be developed in terms of (and deviations from) asymptotic equipartition. Let us review. Consider a given process P and let $A^{l}$ denote the set of its possible length-*ℓ* realizations. Then, for an arbitrary 0<ϵ≪1, the process’ *typical set* is as follows:

(5) $$\begin{array}{c} A_{\epsilon }^{l}\;\equiv \;\left\{w:2^{-l(h_{\mu }+\epsilon )}\leq P\left(w\right)\leq 2^{-l(h_{\mu }-\epsilon )},w\in A^{l}\right\}\;. \end{array}$$

This set consists of realizations whose probability scales with the process’ entropy rate [1,30,31]. Moreover, the *Shannon–McMillan–Breiman theorem* [7,32,33] gives the probability of observing one of these realizations. That is, for a given ϵ≪1, sufficiently large $l^{*}$, and $w\in A^{l}$,

(6) $$\begin{array}{c} P(w\in A_{\epsilon }^{l})\geq 1-\epsilon \;, \end{array}$$

for all $l\geq l^{*}$. There are three lessons:

1. Asymptotic equipartition: Equation (5) says that the probability of each sequence in the typical set decays at approximately the same rate.
2. Typicality: Equation (6) says that for large *ℓ* the probability of observing some typical realization goes to one. Overwhelmingly, they are what one observes.
3. Fluctuations: Conversely, the probability of observing realizations outside the typical set is close to zero. These are the sets of rare sequences or what we call *fluctuations*.

**Figure 5:**
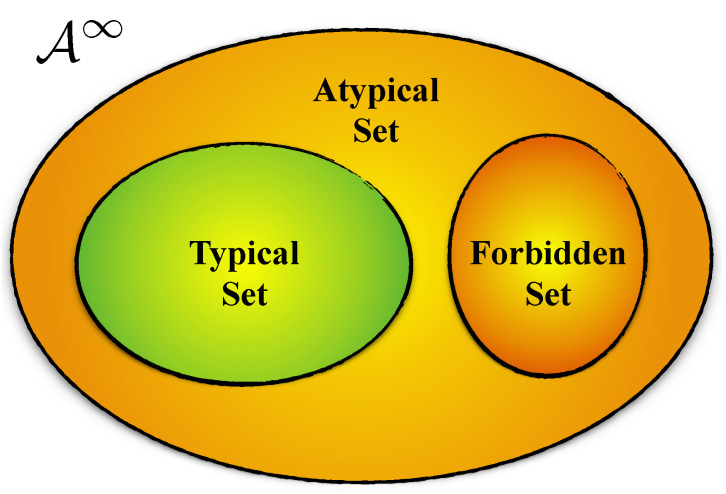
For a given process, the space $A^{\infty }$ of all sequences is partitioned into forbidden sequences, sequences in the typical set, and sequences neither forbidden nor typical—the *atypical* or rare sequences.

As a result, sequences generated by a stationary ergodic process fall into one of three partitions, as depicted in Figure 5. The first contains sequences that are never generated; they fall in the *forbidden set*. The second is the typical set. And, the last contains sequences in a family of *atypical sets*—realizations that are rare to different degrees. Appendix A illustrates these for a Biased Coin Process.

What does this partitioning say about fluctuations in thermodynamic functioning? Recall the functionings identified by the IPSL, as laid out in Table 1. That is, for a given input process, transducer, and temperature, thermodynamic functionality is controlled by three quantities: the average work 〈W〉 generated by the transducer when it operates on the input process, the Shannon entropy $h_{\mu }$ of the input process, and the Shannon entropy $h_{\mu }^{\prime }$ of output process.

Appendix B proves that the difference between average work 〈W(l)〉 over all sequences and that ${\langle W\left(l\right)\rangle }_{TS}$ defined for typical set is small for sufficiently large *ℓ*. For all practical purposes, they are equal. This, together with recalling that ${\langle W\left(l\right)\rangle }_{TS}$ is an unweighted average of works f(w) for $w\in A_{\epsilon }^{l}$, provides an operational interpretation of works used in typical-set-defined functionality.

Similarly, Appendix C proves that the average generated information, when the transducer is fed the whole set, is essentially equal to the average information generated when the transducer is fed the typical set without probability weights.

From Equation (5), it is also clear that the Shannon entropy rate of the input process is also a function of the typical set. This demonstrates that all three quantities—〈W〉, $h_{\mu }$, and $h_{\mu }^{\prime }$—effectively measure properties of the typical set and not of other (atypical) partitions. Recalling that these three quantities also determine the thermodynamics via the IPSL functionality highlights that the previously defined functionality is limited. Next, we remove this limitation, extending the thermodynamic functionality to the whole set of partitions.

## 8. Functioning Outside Typical Realizations

The last section established that the average work 〈W(l)〉 and input and output entropy rates can be used, for l≫1, to identify the system functionality for typical realizations. At last, “typical” has a precise operational meaning. Moreover, as l→∞, the fraction of information available about the functionality of realizations outside the typical set vanishes. Since the probability of observing realizations in the typical set at large *ℓ* approaches one, the definition of functionality based on 〈W〉 and the entropies is very useful.

However, one should not forget that this definition is limited, applying only to one particular subset of realizations. As a result, the associated definition of functionality gives an incomplete picture. How incomplete? Note that the size of the typical set grows like $2^{h_{\mu }l}$ and the size of the whole set, excluding forbidden realizations, grows as $2^{hl}$, where *h* is the input process’ *topological entropy* [34]. Generally, $h>h_{\mu }$ (except for the special class of maximum-entropy processes, which we do not consider directly). And so, the relative size of the typical set shrinks exponentially with *ℓ* as $2^{-(h-h_{\mu })l}$, even though the probability of observing typical realizations converges to one. The lesson is that, at finite *ℓ*, only considering the typical set misses exponentially many—$2^{-(h-h_{\mu })l}$—possibly functional, observable realizations. With this as motivation, we are ready to define functionality for all realizations—typical and atypical—allowing one to describe “nearby” functionalities that arise during fluctuations out of the typical set. The goal is a complete picture of functional fluctuations for finite, but long, realizations.

What engine functionalities do atypical realizations support? The very first step is to partition the set $A^{l}$ of all possible realizations into the subsets of interest. How? We must find a suitable, physically relevant parametrization of realization subsets. We call the collections a process’ *atypical sets*, using degrees of typicality as a parameter.

A key step in the last section was to realize that functionality is defined for unweighted sets of realizations. Recalling Equation (5)’s definition of typical set, the normalized minus logarithm of probabilities—effectively a decay rate—of all the words in the typical set is sandwiched by small deviations (±ϵ) from the Shannon entropy rate:

$$\begin{array}{c} h_{\mu }-\epsilon \leq -\frac{1}{l}\log_{2}P\left(w\right)\leq h_{\mu }+\epsilon \;. \end{array}$$

This is the main reason why ${\langle W\rangle }_{TS}$ is approximately the unweighted average work and, consequently, why functionality is operationally defined for an unweighted set—the typical set. This provides an essential clue as to how to partition the set $A^{l}$ of all possible realizations, at fixed length *ℓ*.

We collect all the realizations with the same probability in the same subset, labeling it with a decay rate denoted *u*:

(7) $$\begin{array}{cc} \Lambda_{u,l} & =\left\{w:-\frac{\log_{2}P\left(w\right)}{l}=u,\;w\in A^{l}\right\}\;. \end{array}$$

Defining $\Lambda_{u}=\lim_{n\to \infty }\Lambda_{u,n}$, it is easy to show that $\Lambda_{u}\subset A^{\infty }$ are disjoint and partition $A^{\infty }$.

Technically, this definition for the (parametrized) subsets of interest is necessary to guarantee consistency with the previously defined typical-set notion of functionality.

**Figure 6:**
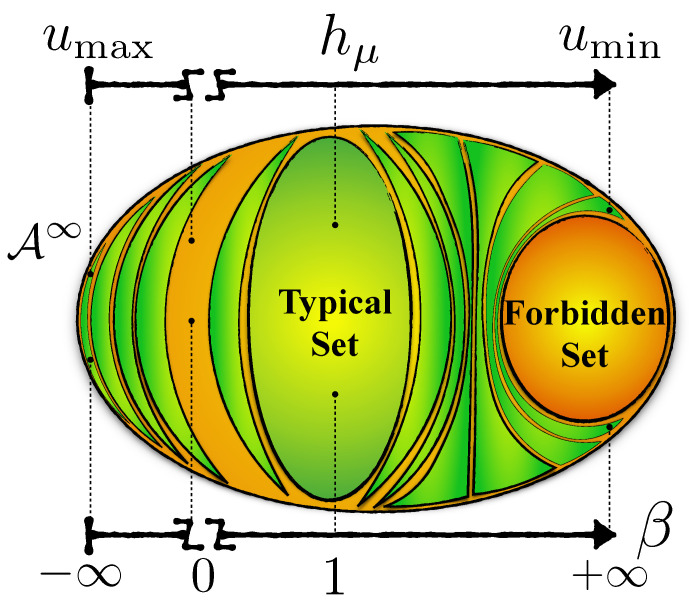
Space $A^{\infty }$ of all sequences partitioned into subsets $\Lambda_{u}$—isoenergy-density or equal probability-decay-rate bubbles—in which all sequences in the same $\Lambda_{u}$ have the same energy density *u*. The typical set is one such bubble with energy equal to Shannon entropy rate: $u=h_{\mu }$. Another important class is the forbidden set, in which all sequences do not occur. The forbidden set can also be interpreted as the subset of sequences with infinite positive energy. By applying the map $B_{\beta }$ to the process and changing β continuously from −∞ to +∞ (excluding β=0) one can generate any atypical class of interest $\Lambda_{u}^{P}$. β→−∞ corresponds to the most probable sequences with the largest energy density $u_{\max}$, β=1 corresponds to the typical set, and β→+∞ corresponds to the least probable sequences with the smallest energy density $u_{\min}$. (Reprinted with permission from Ref. [36]).

The parameter *u*, considered as a random variable, is sometimes called a *self process* [35]. Figure 6 depicts these subsets as “bubbles” of equal decay rate. Equation (5) says the typical set is that bubble with a decay rate equal to the process’ Shannon entropy rate: $u=h_{\mu }$. All the other bubbles contain rare events, some rarer than others, in the sense that they exhibit faster or slower probability decay rates.

The previous section shows that for l≫1 the averaging operator 〈·〉 yields a statistic essentially about the typical set. Now, consider the situation in which we are interested in the functionality of another subset with decay rate $u\neq h_{\mu }$. How can we use the same operator to find the functionality arising from this subset?

If someone presents us with another process $P^{u}$ whose typical set is $\Lambda_{u}$ and we feed this new process into the system, instead of the original input process, then the operator can be used to identify the functionality of realizations in $\Lambda_{u}$. Now, the question comes up as to whether this process exists at all and, if so, can we find it?

The answer to the first question is positive, since we made certain to define the atypical subsets in a way consistent with the definition of the typical set. And, by definition, all the sequences in the subset $\Lambda_{u}$ have the same decay rate.

The answer to the second question is also positive. As argued earlier, we use hidden Markov models (HMMs) as our choice of process representation. Denote process P’s HMM by $M\left(P\right)=\left\{𝓢,\{T^{\left(x\right)}:x\in A\}\right\}$. The question is now framed, What is $M(P^{u})$?

To answer, define a new process $P_{\beta }$ with HMM $M\left(P_{\beta }\right)=\left\{𝓢,\{S_{\beta }^{\left(x\right)},x\in A\}\right\}$. Notice both $M(P_{\beta })$ and M(P) have the same states 𝓢 and same alphabet A. The substochastic matrices of $M(P_{\beta })$ are related to the substochastic matrices of M(P) via the following construction [36,37]:

1. Pick a β∈R.
2. For each x∈A, construct a new matrix $T_{\beta }^{\left(x\right)}$ for which ${\left(T_{\beta }^{\left(x\right)}\right)}_{ij}={\left(T^{\left(x\right)}\right)}_{ij}^{\beta }$.
3. Form the matrix $T_{\beta }=\sum_{x\in A}T_{\beta }^{\left(x\right)}$.
4. Calculate $T_{\beta }$’s maximum eigenvalue ${\hat{\lambda }}_{\beta }$ and corresponding right eigenvector ${\hat{r}}_{\beta }$.
5. For each x∈A, construct new matrices $S_{\beta }^{\left(x\right)}$ for which
  $$\begin{array}{c} {\left(S_{\beta }^{\left(x\right)}\right)}_{ij}=\frac{{\left(T_{\beta }^{\left(x\right)}\right)}_{ij}{\left({\hat{r}}_{\beta }\right)}_{j}}{{\hat{\lambda }}_{\beta }{\left({\hat{r}}_{\beta }\right)}_{i}}\;. \end{array}$$

We defined the new process $P_{\beta }$ by constructing its HMM. We now use the latter to produce an atypical set of interest, say, $\Lambda_{u,l}$.

**Theorem** **1.** *Within the new process $P_{\beta }$, in the limit l→∞, the probability of generating realizations from the set $\Lambda_{u,l}$ converges to one:*

$$\begin{array}{c} \lim_{l\to \infty }{\mathrm{Pr}}_{\beta }\left(\Lambda_{u,l}\right)=1\;, \end{array}$$

*where the energy density is as follows:*

(8) $$\begin{array}{c} u=\beta^{-1}\left(h_{\mu }\left(P_{\beta }\right)-\log_{2}{\hat{\lambda }}_{\beta }\right)\;. \end{array}$$

*Additionally, in the same limit, the process $P_{\beta }$ assigns equal energy densities (probability decay rates) to all $w\in \Lambda_{u,l}$.*

**Proof.**  See Ref. [38]. □

In this way, for large *ℓ* the process $P_{\beta }$ typically generates realizations in the set $\Lambda_{u,l}$ and with the specified energy *u*. The process $P_{\beta }$ is variously called the *auxiliary*, *driven*, or *effective process* [39,40,41].

Using Equation (8), one can show that for any *u* there exists a unique and distinct β∈R and, moreover, that *u* is a decreasing function of β. And so, we can equivalently denote the process $P_{\beta }$ by $P^{u}$. More formally, every word in $\Lambda_{u}$ with probability measure one is in the typical set of process $P_{\beta }$. Thus, sweeping β∈[−∞,∞] controls which subsets (atypical sets) outside the typical set we focus on. And, applying the operator 〈·〉 determines the engine functionality for realizations in that subset, as we now show.

## 9. Functional Fluctuations

Let us draw out the consequences and applications of this theory of functional fluctuations. First, we ground the results by identifying the range of functionality that arises as an information ratchet (introduced earlier) operates. Then, we turn to showing how to calculate the probability of its fluctuating functionalities.

### 9.1. An Information Ratchet Fluctuates

Recall the information ratchet introduced in Section 4, but now set its Markov dynamic parameters p=0.2 and q=0.6 and put it in contact with an information reservoir that generates IID symbol sequences with bias b=0.9. Operating the input reservoir for a sufficiently long period, with high probability, we observe a sequence that has nearly 90% 0 s in it. Using Equations (3) and (4), we see positive work 〈W〉>0 and positive entropy production $h_{\mu }^{\prime }-h_{\mu }>0$. Then, according to the IPSL functionalities in Table 1, the ratchet typically operates as an engine.

**Figure 7:**
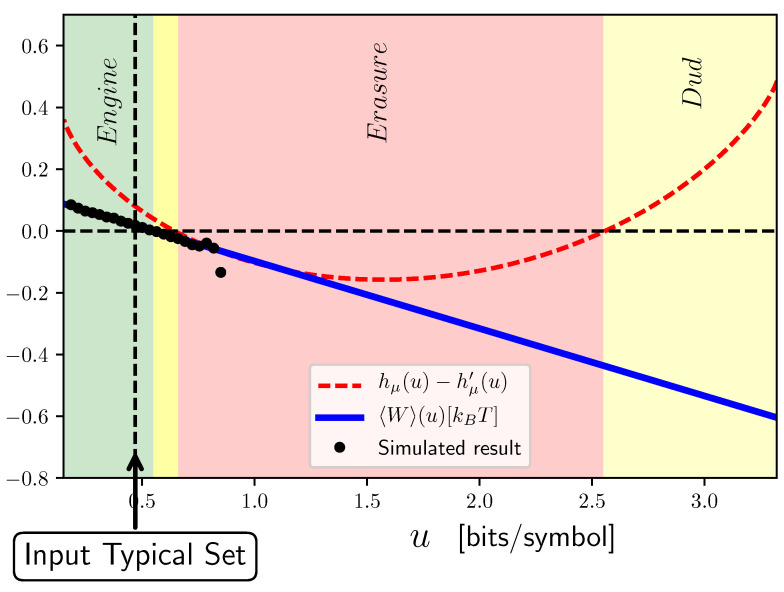
Average work 〈W〉(u) (blue line) and difference $h_{\mu }^{\prime }-h_{\mu }$ between output and input Shannon entropy rate, respectively, (red dashed line) versus decay rate *u* for different atypical sets (fluctuations). In this, information transducer with parameters p=0.2 and q=0.6 is driven by an IID input source with bias b=0.9. Table 1 has been used to identify functionality of different fluctuations subsets: engine (green), eraser (red), and dud (yellow, two regions).

What thermodynamic functionalities occur when the input fluctuates outside the typical set? Sweeping β controls which subsets outside the typical set are expressed and, consequently, which fluctuation subsets are accessible. Recall that the input process is specified by the unifilar HMM in Figure 3a. For this input, as a result of the ratchet design, $M(P_{\beta })$ is the same as M(P), except that *b* is shifted to $\hat{b}=b^{\beta }/\left(b^{\beta }+{\left(1-b\right)}^{\beta }\right)$. Different sequence–probability decay rates *u* are calculated from Equation (8). Then, feeding the new process to the ratchet, 〈W〉 is calculated from Equation (3), again by changing *b* to $\hat{b}$. Denote this work quantity 〈W〉(u). Figure 7 shows the dissipated work 〈W〉(u) and the difference between the output and input Shannon entropy rate versus the fluctuating decay rate *u*. There are several observations to make, before associating the thermodynamic function.

First, let us locate the input typical set. This occurs at a *u* such that β=1. The figure identifies it with a vertical line, so labeled.

Second, the input process’ ground states occur as β→∞ since *u* is a decreasing function of β. As a consequence of Equation (7), this subset corresponds to the sequence with the highest probability. In this case, this is the all-0 s sequence with $u_{\min}=-\log_{2}\left(b\right)≃0.152$. The other extreme is at $u_{\max}$, corresponding to the lowest-probability, allowed sequence. This is the all-1 s sequence with $u_{\max}=-\log_{2}\left(1-b\right)≃3.32$. Note that there is only a single sequence associated with $u_{\max}$ and only one with $u_{\min}$.

Third, to complete the task of identifying function, we must determine the average work 〈W〉 as a function of energy *u*. From the figure, we see that the dissipated work 〈W〉 is linear in the decay rate *u*. Appendix D derives this and also shows that the maximum work over all subsets—all β or all allowed decay rates *u*—is independent of the input process bias. This is perhaps puzzling as bias clearly controls the ratchet’s thermodynamic behavior. Thus, assuming an IID input, the maximum work is a property of the ratchet itself and not the input—the maximum work playing a role rather analogous to how Shannon’s channel capacity is a channel property.

To better understand how the ratchet operates thermodynamically, consider the ground state of the input process, which as just noted has only a single member, the all-0 sequence with zero entropy rate $h_{\mu }=0$. If we feed this sequence into the ratchet, the ratchet adds stochasticity which appears in the output sequence. The first 0 fed to the ratchet leads to a 0 on the output. For the next 0 fed in, with probability *p* the ratchet outputs 1 and with probability 1−p it outputs 0. The entropy rate of the output sequence then is $h_{\mu }^{\prime }=\frac{1}{2}H\left(p\right)≃0.36$.

To generate this sequence, we simply use the ϵ-machine in Figure 3 with b=1. With this biased process as input, using Equation (3), we find $\langle W\rangle \left(u_{\min}\right)≃0.0875>0$. Table 1 then tells us that if we feed the ground state of the input process to the ratchet, it functions as an engine. At the other extreme, $U_{\max}$, the only fluctuation subset member is the all-1 s sequence with $h_{\mu }=0$. Again, the ratchet adds stochasticity and the output has $h_{\mu }^{\prime }=\frac{1}{2}H\left(q\right)≃0.485$. To generate this input sequence, we simply use the ϵ-machine in Figure 3 with b=0. With this process as an input, we use Equation (3) again and find negative work $\langle W\rangle \left(U_{\max}\right)≃-0.6$. Table 1 now tells us that feeding in this extreme sequence (input fluctuation) the ratchet functions as a dud.

Overall, Table 1 allows one to identify the regimes of *u* associated with distinct thermodynamic functionality. These are indicated in Figure 7 with the green region corresponding to engine functioning, red to eraser functioning, and yellow to dud. We conclude that the ratchet’s thermodynamic functioning depends substantially on fluctuations and so will itself fluctuate over time. In particular, engine functionality occurs only at relatively low input fluctuation energies, seen on Figure 7’s left side, and encompasses the typical set, as a consequence of our design. Rather nearby the engine regime, though, is a narrow one of no functioning at all—a dud. In fact, though the ratchet was designed as an engine, we see that, over most of the range of fluctuations, with the given parameter setting, the ratchet operates as an eraser.

### 9.2. Probable Functional Fluctuations

In this way, we see that typical-set functionality can be extended to all input realizations—that is, to all fluctuation subsets. The results give insight into the variability in thermodynamic function and a direct sense of its robustness or lack thereof. Now, we answer two questions that are particularly pertinent in the present setting of events (sequences) whose probabilities decay exponentially fast and so may be practically never observed. How probable are fluctuations in thermodynamic functioning? And, the related question, how probable are each of the fluctuation subsets? Exploring one example, we will show that the functional fluctuations are, in fact, quite observable not only with short sequences, perhaps expectedly, but also over relatively long sequences, such as l=100.

The second question calls for determining $P(w\in \Lambda_{u,l})$. However, in the large-*ℓ* limit, this quantity vanishes. So, it is rather more natural to ask *how* it converges to zero. Since we are considering ergodic stationary processes, we can apply the large deviation principle: the probability of every subset $\Lambda_{u,l}$ vanishes exponentially with *ℓ*. However, each subset $\Lambda_{u,l}$ has a different exponent which is the subset’s *large deviation rate* [35]:

$$\begin{array}{c} I\left(u\right)=\lim_{l\to \infty }-\frac{1}{l}\log_{2}P\left(w\in \Lambda_{u,l}\right)\;. \end{array}$$

Since all these *w* have the same probability decay rate *u*, P(w) decomposes to two components. The first gives the number $|\Lambda_{u,l}|$ of sequences in the subset and the second the probability $2^{-lu}$ of individual sequences. That is,

$$\begin{array}{cc} I(u) & =\lim_{l\to \infty }-\frac{1}{l}\log_{2}P\left(w\in \Lambda_{u,l}\right)\\ \; & =\lim_{l\to \infty }-\frac{1}{l}\log_{2}\left(\right|\Lambda_{u,l}\left|2^{-lu}\right)\\ \; & =u-\lim_{l\to \infty }\frac{1}{l}\log_{2}\left(\right|\Lambda_{u,l}\left|\right)\;. \end{array}$$

The size of the subsets also grows exponentially with *ℓ*, each subset with a different exponent. To monitor this, we define a new function:

$$\begin{array}{c} S\left(u\right)=\lim_{l\to \infty }\frac{1}{l}\log_{2}\left|\Lambda_{u,l}\right|\;. \end{array}$$

Previously, we showed that $S\left(u\right)=h_{\mu }\left(P_{\beta }\right)$, where $h_{\mu }\left(P_{\beta }\right)$ is $P_{\beta }$ Shannon entropy and $u=\beta^{-1}\left(h_{\mu }\left(P_{\beta }\right)-\log_{2}{\hat{\lambda }}_{\beta }\right)$ from Equation (8) [38]. These results allow one to calculate I(u) for any subset using the following expressions:

$$\begin{array}{cc} I(u) & =\left(\beta^{-1}-1\right)h_{\mu }\left(P_{\beta }\right)-\beta^{-1}\log_{2}{\hat{\lambda }}_{\beta }\;\mathrm{and}\\ u & =\beta^{-1}\left(h_{\mu }\left(P_{\beta }\right)-\log_{2}{\hat{\lambda }}_{\beta }\right)\;. \end{array}$$

**Figure 8:**
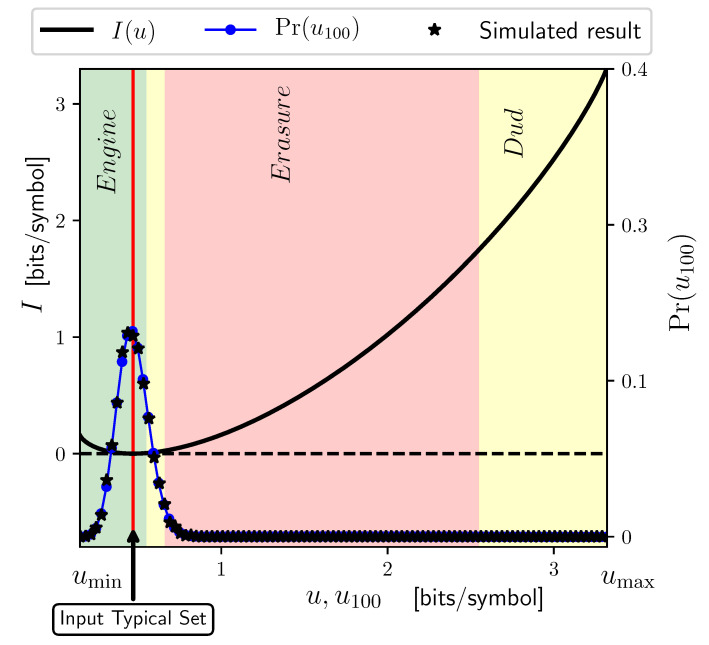
Probability of fluctuations in thermodynamic functioning: large-deviation rate function I(u) (solid black line) and the theoretically predicted probability $\mathrm{Pr}(u_{100})$ of fluctuation subsets for length l=100 input realizations (dotted–solid blue line). Star tokens denote estimates from numerical simulation which validate the analytical results due to their close fit.

Figure 8 plots I(u) for our example information ratchets. As with the previous figure, when realizations from the typical set are fed in, the transducer functions as an engine. We now see that the typical set has a zero large deviation rate. That is, in the limit of infinite length, the probability of observing realizations in the typical set goes to one. In terms of thermodynamic functioning, the transducer operates as an engine over long periods with probability one. Complementarily, in the infinite length limit, the probability of the other “fluctuation” subsets vanishes.

In reality, though, one only observes finite-length sequences. And so, the operant question here is, are functional fluctuations observable at finite lengths? As we alluded to earlier, the expectation is that short sequences should enhance their observation.

Consider the input process in Figure 3a and assume the input’s realization length is l=100. We have $2^{100}$ distinct input sequences that are partitioned into 101 fluctuation subsets with different energy densities—subsets of sequences with *ℓ* 0 s and 100−l 1 s for l=0,1,…,100. Let us calculate the probability of each of these fluctuation subsets occurring analytically. The probability of each versus its energy is shown in Figure 8 as the blue dotted line. To distinguish it from the energy density of fluctuation subsets at infinite length we label the energy density of each of these sets with $u_{100}$; the index 100 reminds us that we are examining input sequences of length l=100. There are 101 blue points on the figure, each representing one of the fluctuation subsets. (Most are obscured by other tokens, though.) If we feed the first 13 of the 101 fluctuation subsets (the first 13 blue points on the left of the figure) to the transducer, it functions as an engine. Summing the probabilities of these engine subsets, we see that the transducer functions as an engine 80% of the time, which is quite probable, even though it operates on sequences of length 100 that are individually highly improbable.

To verify the analytical results, we also performed extensive numerical simulations that drove the ratchet with a sequence of length $l=10^{6}$. We divided the input sequence into time intervals of length 100 and estimated the generated work and other observables, such as energy, during each interval. The star tokens in Figure 7 show the estimated average work in each interval with a decay rate *u* versus the decay rate itself. The numerical estimates agree closely with the analytical result. Figure 8 also shows the probabilities of each of these atypical subsets estimated from the simulations, which also validates the analytical results.

Let us return to the remaining question: how probable are fluctuations in thermodynamic functioning? The answer is given by the large deviation rate for 〈W〉(u). Since 〈W〉 is a function of *u*, one can use the contraction principle [35] and relate the large deviation rate of 〈W〉(u) in terms of a large deviation rate of *u* via the following:

$$\begin{array}{c} \tilde{I}\left(y=\langle W\rangle \left(u\right)\right)=\min_{u:y=\langle W\rangle (u)}I\left(u\right)\;. \end{array}$$

Since 〈W〉(u) is a one-to-one function, the minimization above may be removed.

## 10. Discussion

### 10.1. Related Work

The new results here on memoryful information engines are also complementary to previous studies of fluctuations in the efficiency of a nanoscale heat engine [42,43,44], a particular form of information engine.

### 10.2. Relation to Fluctuation Theorems

To head off confusion, and anticipate a key theme, note that the “statistical fluctuation” above differs importantly from the sense used to describe variations in mesoscopic quantities when controlling small-scale thermodynamic systems. This latter sense is found in the recently famous fluctuation theorem for the probability of positive and negative entropy production ΔS during macroscopic thermodynamic manipulations [45,46,47,48,49,50,51]:

$$\begin{array}{c} \frac{\mathrm{Pr}(\Delta S)}{\mathrm{Pr}(-\Delta S)}=e^{\Delta S}\;. \end{array}$$

Both kinds of fluctuation are ubiquitous, often dominating equilibrium finite-size systems and finite and infinite nonequilibrium steady-state systems. Differences acknowledged, there are important connections between statistical fluctuations in microstates observed in steady state and fluctuations in thermodynamic variables encountered during general control: for one, they are deeply implicated in expressed thermodynamic function. Is a system operating as an engine—converting thermal fluctuations to useful work—or as an eraser—depleting energy reservoirs to reduce entropy—or not functioning at all?

## 11. Conclusions

We synthesized statistical fluctuations—as entailed in Shannon’s Asymptotic Equipartition Property [1] and large deviation theory [35,52,53]—and functional thermodynamics—as determined using the new informational second law [3]—to predict spontaneous variations in thermodynamic functioning. In short, there is simultaneous, inherently parallel, thermodynamic processing that is functionally distinct and possibly in competition. This strongly suggests that, even when in a nonequilibrium steady state, a single nanoscale device or biomolecule can be both an engine and an eraser. And, we showed that these functional fluctuations need not be rare. This complements similar previous results on fluctuations in small-scale engine efficiency [42,43,54]. The conclusion is that functional fluctuations should be readily observable and the prediction experimentally testable.

A main point motivating this effort was to call into question the widespread habit of ascribing a single functionality to a given system and, once that veil has lifted, to appreciate the broad consequences. To drive them home, since biomolecular systems are rather like the information ratchet here, they should exhibit measurably different thermodynamic functions as they behave. If this prediction holds, then the biological world is vastly richer than we thought and it will demand of us a greatly refined vocabulary and greatly improved theoretical and experimental tools to adequately probe and analyze this new modality of parallel functioning.

That said, thoroughness forces us to return to our earlier caveat (Section 9) concerning not conflating various “temperatures”. If we give the input information reservoir and the output information reservoir physical implementations, then the fluctuation indices $U_{\mathrm{in}}$ and $U_{\mathrm{out}}$ take on thermal physical meaning and so can be related to the ratchet’s thermodynamic temperature *T*. Doing so, however, would take us too far afield here, but it will be necessary for a complete understanding.

Looking forward, there are many challenges. First, note that technically speaking we introduced a fluctuation theory for memoryful stochastic transducers, but by way of the example of Ref. [3]’s information ratchet. A thoroughgoing development must be carried out in much more generality using the tools of Refs. [29,38], if we are to fully understand the functionality of thermodynamic processes that transform inputs to outputs, environmental stimulus to environmental action.

Second, the role of the Jarzynski–Crooks theory for fluctuations in thermodynamic observables needs to be made explicit and directly related to statistical fluctuations, in the sense emphasized here. One reason is that their theory bears directly on controlling thermodynamic systems and the resulting macroscopic fluctuations. To draw the parallel more closely, following the fluctuation theory for transitions between nonequilibrium steady states [55], we could drive the ratchet parameters *p* and *q* and input bias *b* between different functional regimes and monitor the entropy production fluctuations to test how the theory fares for memoryful processes. In any case, efficacy in control will also be modulated by statistical fluctuations.

Not surprisingly, there is much to do. Let us turn to a larger motivation and perhaps larger consequences to motivate future efforts.

As just noted, fluctuations are key to nanoscale physics and molecular biology. We showed that fluctuations are deeply implicated both in identifying thermodynamic function and in the very operation of small-scale systems. In fact, fluctuations are critical to life—its proper and robust functioning. The perspective arising from parallel thermodynamic function is that, rather than fluctuations standing in contradiction to life processes, potentially corrupting them, there may be a positive role for fluctuations and parallel thermodynamic functioning. Once that is acknowledged, it is a short step to realize that biological evolution may have already harnessed them to good thermodynamic effect. Manifestations are clearly worth looking for.

It now seems highly likely that fluctuations engender more than mere health and homeostasis. It is a commonplace that biological evolution is nothing, if not opportunistic. If so, then it would evolve cellular biological thermodynamic processes that actively leverage fluctuations. Mirroring Maxwell’s Demon’s need for fluctuations to operate, biological evolution itself advances only when there are fluctuations. For example, biomolecular mutation processes engender a distribution of phenotypes and fitnesses; fodder for driving selection and so evolutionary innovation. This, then, is *Darwin’s Demon*—a mechanism that ratchets in favorable fluctuations for a positive thermodynamic and then positive survival benefit. The generality of results and methods here give new insight into thermodynamic functioning in the presence of fluctuations that should apply at many different scales of life, including its emergence and evolution.

## Figures and Tables

**Table 1:** Thermodynamic functionings for information engines, as determined by the information processing second law of Equation (1).

Modality	Function	Net Work	Net Computation
Engine	Extracts high-entropy energy from the thermal reservoir, converts it into low-entropy work by randomizing output	〈W〉>0	$h_{\mu }^{\prime }-h_{\mu }>0$
Eraser	Uses low-entropy energy from work reservoir to reduce input randomness, exhausting high-entropy energy to thermal reservoir	〈W〉<0	$h_{\mu }^{\prime }-h_{\mu }<0$
Ineffective randomizer	Wastes stored work (low-entropy energy) to randomize output	〈W〉<0	$h_{\mu }^{\prime }-h_{\mu }>0$

## Data Availability

The original contributions presented in the study are included in the article, further inquiries can be directed to the corresponding author.

## Appendix A. Typical Set for a Biased Coin

What is $A_{\epsilon }^{n}$ for a biased coin with bias *b*? The typical set is defined by the following:

$$\begin{array}{c} A_{\epsilon }^{n}=\left\{w\in A^{n}:2^{-n(h_{\mu }+\epsilon )}\leq \mathrm{Pr}\left(w\right)\leq 2^{-n(h_{\mu }-\epsilon )}\right\}\;. \end{array}$$

The probability of a biased coin generating a particular sequence *w* with *k* heads is $b^{k}{\left(1-b\right)}^{\left(n-k\right)}$. And so, for *w* to be in the typical set, we must have the following:

$$\begin{array}{c} lb-\frac{n\epsilon }{\log\frac{b}{1-b}}\leq k\leq nb+\frac{n\epsilon }{\log\frac{b}{1-b}}\;. \end{array}$$

Since *k* is an integer,

$$\begin{array}{c} \lceil nb-\frac{n\epsilon }{\log\frac{b}{1-b}}\rceil \leq k\leq \lfloor nb+\frac{n\epsilon }{\log\frac{b}{1-b}}\rfloor \;. \end{array}$$

For example, in the case where n=1000, b=0.6, and ϵ=0.01, we have the following:

$$\begin{array}{c} 582\leq k\leq 617\;. \end{array}$$

This means that those length n=1000 sequences with 582 to 617 heads are in the typical set.

## Appendix B. Work Bounds

The average work for finite length *ℓ* is as follows:

(A1) $$\begin{array}{c} \langle W\left(l\right)\rangle =\frac{1}{l}\sum_{w\in A^{l}}P\left(w\right)f\left(w\right)\;. \end{array}$$

Recall that f(w) is the average work generated by the transducer when fed the word *w*; see Figure 2.

Now, let us say we are only interested in the engine’s functionality when operating on sequences in a particular partition—those in the typical set. To determine the functionality, we first define a new probability distribution for the typical set:

$$\begin{array}{c} \tilde{P(w)}=\left\{\begin{array}{cc} P\left(w\right)/\;\;\;\;\sum_{w\in A_{\epsilon }^{l}}P\left(w\right)\; & \;w\in A_{\epsilon }^{l}\\ 0 & \;w\notin A_{\epsilon }^{l} \end{array}\right.\;. \end{array}$$

Using it, we define a new average work for finite *ℓ* sequences in the typical set:

$$\begin{array}{c} {\langle W\left(l\right)\rangle }_{TS}=\frac{1}{l}\sum_{w\in A^{l}}\tilde{P(w)}f\left(w\right)\;. \end{array}$$

There are two important observations. First, this statistic gives no information about works generated by sequences outside of $A_{\epsilon }^{l}$, since the probability distribution vanishes there. Second, for every pair of typical sequences $w_{1},w_{2}\in A_{\epsilon }^{l}$,

$$\begin{array}{c} \tilde{P(w_{1})}≃\tilde{P(w_{2})}\;, \end{array}$$

since Equation (5) bounds the sequences’ probabilities: $2^{-l(h_{\mu }+\epsilon )}\leq \mathrm{Pr}\left(w\right)\leq 2^{-l(h_{\mu }-\epsilon )}$. Effectively, ${\langle W\left(l\right)\rangle }_{TS}$ is an unweighted average over f(w)s for the words in the typical set.

Now, consider Equation (A1) and decompose its righthand side into two parts: the share of typical sequences and the share of atypical sequences:

(A2) $$\begin{array}{c} \sum_{w\in A^{l}}P\left(w\right)f\left(w\right)=\sum_{w\in A_{\epsilon }^{l}}P\left(w\right)f\left(w\right)+\sum_{w\notin A_{\epsilon }^{l}}P\left(w\right)f\left(w\right)\;. \end{array}$$

The second term is bounded from above:

$$\begin{array}{cc} \sum_{w\notin A_{\epsilon }^{l}}P\left(w\right)f\left(w\right)\; & \leq \left(\sum_{w\notin A_{\epsilon }^{l}}P\left(w\right)\right)\max\left\{f\left(w\right):w\notin A_{\epsilon }^{l}\right\}\\ \; & \leq \epsilon \times \max\{f\left(w\right):w\notin A_{\epsilon }^{l}\}\;. \end{array}$$

f(w), the work generated by any length-*ℓ* sequence $w\in A^{l}$, is also bounded:

$$\begin{array}{c} l\alpha_{\min}\leq f\left(w\right)\leq l\alpha_{\max}\;, \end{array}$$

where $\alpha_{\min}$ and $\alpha_{\max}$ are the minimum and maximum one-shot works, respectively. Here, $\alpha_{\max}>0$ and $\alpha_{\min}<0$, which are due to the finiteness of energy for the machine states and coupled input symbol states. As a result, we have:

$$\begin{array}{c} \sum_{w\notin A_{\epsilon }^{l}}P\left(w\right)f\left(w\right)\leq l\epsilon \alpha_{\max}\;. \end{array}$$

Similarly, it has a lower bound:

$$\begin{array}{c} \sum_{w\notin A_{\epsilon }^{l}}P\left(w\right)f\left(w\right)\geq \epsilon l\alpha_{\min}\;. \end{array}$$

Now, we turn to decompose Equation (A2)’s first term into two parts:

(A3) $$\begin{array}{cc} \sum_{w\in A_{\epsilon }^{l}}P\left(w\right) & f\left(w\right)=\sum_{w\in A_{\epsilon }^{l}}\frac{P(w)}{\sum_{w\in A_{\epsilon }^{l}}P\left(w\right)}f\left(w\right)\\ \; & +\sum_{w\in A_{\epsilon }^{l}}\left(P\left(w\right)-\frac{P(w)}{\sum_{w\in A_{\epsilon }^{l}}P\left(w\right)}\right)f\left(w\right)\;. \end{array}$$

The second term in Equation (A3) can be written as follows:

$$\begin{array}{cc} \sum_{w\in A_{\epsilon }^{l}}\; & \left(P\left(w\right)-\frac{P(w)}{\sum_{w\in A_{\epsilon }^{l}}P\left(w\right)}\right)f\left(w\right)=\\ \; & \;\left(1-\frac{1}{\sum_{w\in A_{\epsilon }^{l}}P\left(w\right)}\right)\sum_{w\in A_{\epsilon }^{l}}P\left(w\right)f\left(w\right)\;, \end{array}$$

To go further one must note that the coefficient of the sum on the righthand side is negative. As a result,

$$\begin{array}{cc} \sum_{w\in A_{\epsilon }^{l}}\; & \left(P\left(w\right)-\frac{P(w)}{\sum_{w\in A_{\epsilon }^{l}}P\left(w\right)}\right)f\left(w\right)\\ \; & \leq \left(1-\frac{1}{\sum_{w\in A_{\epsilon }^{l}}P\left(w\right)}\right)\min\left\{f\left(w\right):w\notin A_{\epsilon }^{l}\right\}\sum_{w\in A_{\epsilon }^{l}}P\left(w\right)\\ \; & \leq \left(1-\frac{1}{\sum_{w\in A_{\epsilon }^{l}}P\left(w\right)}\right)l\alpha_{\min}\sum_{w\in A_{\epsilon }^{l}}P\left(w\right)\\ \; & \leq \left(1-\frac{1}{1-\epsilon }\right)l\alpha_{\min}\;. \end{array}$$

This gives an upper bound on the second term in Equation (A3).

Similarly, one can give it a lower bound:

$$\begin{array}{c} \sum_{w\in A_{\epsilon }^{l}}\left(P\left(w\right)-\frac{P(w)}{\sum_{w\in A_{\epsilon }^{l}}P\left(w\right)}\right)f\left(w\right)\geq \left(1-\frac{1}{1-\epsilon }\right)l\alpha_{\max}\;. \end{array}$$

Using Equations (A2) and (A3) and these upper and lower bounds, we have the following:

$$\begin{array}{c} \delta_{\min}\leq \langle W\left(n\right)\rangle -{\langle W\left(n\right)\rangle }_{TS}\leq \delta_{\max}\;, \end{array}$$

where

$$\begin{array}{cc} \delta_{\min}\; & =\epsilon \left(\alpha_{\min}-\frac{\alpha_{\max}}{1-\epsilon }\right)\;\mathrm{and}\\ \delta_{\max}\; & =\epsilon \left(\alpha_{\max}-\frac{\alpha_{\min}}{1-\epsilon }\right)\;. \end{array}$$

Recalling that $\alpha_{\max}>0$ and $\alpha_{\min}<0$, we have $\delta_{\max}>0$ and $\delta_{\min}<0$.

Thus, the difference between average work 〈W(l)〉 over all sequences and that ${\langle W\left(l\right)\rangle }_{TS}$ defined for typical set is small for sufficiently large *ℓ*. For all practical purposes, they are equal. This, together with recalling that ${\langle W\left(l\right)\rangle }_{TS}$ is an unweighted average of works f(w) for $w\in A_{\epsilon }^{l}$, provides an operational interpretation of the previously defined functionality.

## Appendix C. Information Bounds

The Shannon entropy rate of the output process for finite length *ℓ* is as follows:

$$\begin{array}{c} \Delta H^{\prime }\left(l\right)=\frac{1}{l}\sum_{w^{\prime }\in A^{l}}P^{\prime }\left(w^{\prime }\right)\log_{2}P^{\prime }\left(w^{\prime }\right)\;. \end{array}$$

Here, P(·) refers to the probability of output sequences under the process generated by the transducer and $w^{\prime }$ is an output sequence. Rewrite the sum in the form

$$\begin{array}{c} \sum_{w^{\prime }\in A^{l}}P^{\prime }\left(w^{\prime }\right)\log_{2}P^{\prime }\left(w^{\prime }\right)=\sum_{w,w^{\prime }\in A^{l}}P\left(w\right)P\left(w^{\prime }|w\right)\log_{2}P^{\prime }\left(w^{\prime }\right)\;, \end{array}$$

where $P(w^{\prime }|w)$ is the conditional probability of the transducer generating output sequence $w^{\prime }$ when reading input *w*. Now, defining

$$\begin{array}{c} g\left(w\right)=\sum_{w^{\prime }\in A^{l}}-P\left(w^{\prime }|w\right)\log_{2}P^{\prime }\left(w^{\prime }\right)\;, \end{array}$$

one writes the Shannon entropy rate in a form paralleling Equation (A1):

(A4) $$\begin{array}{c} \Delta H^{\prime }\left(l\right)=\frac{1}{l}\sum_{w\in A^{l}}P\left(w\right)g\left(w\right)\;, \end{array}$$

where g(w) is the average information generated by the word *w* when passing through the transducer; see Figure 2.

We can also monitor the information generated by feeding in only the typical set with:

$$\begin{array}{c} \Delta H_{TS}^{\prime }\left(l\right)=\frac{1}{l}\sum_{w\in A^{l}}\tilde{P(w)}g\left(w\right)\;. \end{array}$$

Similar to analyzing the generated works, one decomposes the sum in Equation (A4) into two parts:

(A5) $$\begin{array}{c} \sum_{w\in A^{l}}P\left(w\right)g\left(w\right)=\sum_{w\in A_{\epsilon }^{l}}P\left(w\right)g\left(w\right)+\sum_{w\notin A_{\epsilon }^{l}}P\left(w\right)g\left(w\right)\;. \end{array}$$

The second term in Equation (A5) is bounded above:

$$\begin{array}{cc} \sum_{w\notin A_{\epsilon }^{l}}P\left(w\right)g\left(w\right)\; & \leq \left(\sum_{w\notin A_{\epsilon }^{n}}P\left(w\right)\right)\max\left\{g\left(w\right):w\notin A_{\epsilon }^{l}\right\}\\ \; & \leq \epsilon \times \max\{g\left(w\right):w\notin A_{\epsilon }^{l}\} \end{array}$$

From the definition, one sees that there are upper bounds on g(w)<l, where the bound can only be reached when the input is a Fair Coin Process and the transducer maps every word fairly to all possible output sequences. This means the second term in Equation (A5) is bounded from above:

$$\begin{array}{c} \sum_{w\notin A_{\epsilon }^{l}}P\left(w\right)g\left(w\right)\leq \epsilon l\;. \end{array}$$

We can similarly analyze the first term in Equation (A5):

$$\begin{array}{cc} \sum_{w\in A_{\epsilon }^{l}}P\left(w\right)g\left(w\right) & =\sum_{w\in A_{\epsilon }^{l}}\tilde{P(w)}g\left(w\right)\\ \; & \;+\sum_{w\in A_{\epsilon }^{l}}\left(P\left(w\right)-\tilde{P(w)}\right)g\left(w\right)\;. \end{array}$$

The second term here is negative and is bounded from below:

$$\begin{array}{cc} \sum_{w\in A_{\epsilon }^{n}}\; & (P\left(w\right)-\tilde{P(w)})g\left(w\right)=\\ \; & \left(1-\frac{1}{\sum_{w\in A_{\epsilon }^{l}}P\left(w\right)}\right)\sum_{w\in A_{\epsilon }^{l}}P\left(w\right)g\left(w\right)\\ \; & \geq \left(1-\frac{1}{1-\epsilon }\right)\sum_{w\in A_{\epsilon }^{l}}P\left(w\right)g\left(w\right)\\ \; & \geq \left(1-\frac{1}{1-\epsilon }\right)\max\left\{g\left(w\right):w\notin A_{\epsilon }^{l}\right\}\sum_{w\in A_{\epsilon }^{l}}P\left(w\right)\\ \; & \geq \left(\frac{-\epsilon }{1-\epsilon }\right)\max\left\{g\left(w\right):w\notin A_{\epsilon }^{l}\right\}\\ \; & \geq l\left(\frac{-\epsilon }{1-\epsilon }\right)\;. \end{array}$$

Combining these two bounds,

$$\begin{array}{c} \frac{-\epsilon }{1-\epsilon }\leq \Delta H^{\prime }\left(l\right)-\Delta H_{TS}^{\prime }\left(l\right)\leq \epsilon \;, \end{array}$$

one concludes the average generated information, when the transducer is fed the whole set, is essentially equal to the average information generated when the transducer is fed the typical set without probability weights.

## Appendix D. Work Is a Linear Function of Decay Rate

First, let us calculate *u* as a function of β. Recall that they related via $u=\beta^{-1}\left(h_{\mu }\left(P_{\beta }\right)-\log_{2}{\hat{\lambda }}_{\beta }\right)$.

Using step 2 and 3 for the HMM model shown in Figure 3, we have

$$\begin{array}{cc} T_{\beta } & =\left[\begin{array}{ccc} b^{\beta } & \; & {\left(1-b\right)}^{\beta }\\ b^{\beta } & \; & {\left(1-b\right)}^{\beta } \end{array}\right]\;\;. \end{array}$$

Calculating the maximal eigenvalue ${\hat{\lambda }}_{\beta }$, we find the following:

$$\begin{array}{c} \log\lambda_{\beta }=\log_{2}\left(b^{\beta }+{\left(1-b\right)}^{\beta }\right)\;. \end{array}$$

The Shannon entropy of process $P_{\beta }$, $h_{\mu }\left(P_{\beta }\right)$ is equal to the Shannon entropy of the biased coin with bias $\hat{b}=b^{\beta }/(b^{\beta }+{\left(1-b\right)}^{\beta }$:

$$\begin{array}{cc} h_{\mu }\left(P_{\beta }\right)=- & \left(\frac{b^{\beta }}{b^{\beta }+{\left(1-b\right)}^{\beta }}\log_{2}\frac{b^{\beta }}{b^{\beta }+{\left(1-b\right)}^{\beta }}\right.\\ \; & \left.+\frac{{\left(1-b\right)}^{\beta }}{b^{\beta }+{\left(1-b\right)}^{\beta }}\log_{2}\frac{{\left(1-b\right)}^{\beta }}{b^{\beta }+{\left(1-b\right)}^{\beta }}\right)\;. \end{array}$$

It is straightforward, now, to calculate *u* from these:

$$\begin{array}{cc} u & =\frac{-b^{\beta }}{b^{\beta }+{\left(1-b\right)}^{\beta }}\log_{2}\left(b\right)\\ \; & \;\;+\frac{-{\left(1-b\right)}^{\beta }}{b^{\beta }+{\left(1-b\right)}^{\beta }}\log_{2}\left(1-b\right)\;. \end{array}$$

In the next step, we need to calculate the work 〈W〉 from Equation (3) for the input process $P_{\beta }$ by replacing *b* with $\hat{b}$:

$$\begin{array}{c} \langle W\rangle \left(\beta \right)=\frac{k_{B}T}{2}\left(-q\log\left(q/p\right)+q\log\left(1-q\right)+\frac{cb^{\beta }}{b^{\beta }+{\left(1-b\right)}^{\beta }}\right)\;, \end{array}$$

where c=(p+q)log(q/p)+plog(1−p)−qlog(1−q). Now it is easy to see that:

$$\begin{array}{cc} \langle W\rangle \left(u\right)=\frac{k_{B}T}{2}\; & \left(-q\log\left(\frac{q}{p}\right)+q\log\left(1-q\right))\right.\\ \; & \left.+c\frac{u+\log(1-b)}{\log(1-b)-\log(b)}\right)\;. \end{array}$$

It is also easy to see that:

$$\begin{array}{cc} W_{\max} & =\max_{\beta }\langle W\rangle \\ \; & =\left\{\begin{array}{cc} W_{\max}^{-}\equiv k_{B}T\left(-q\log(p/q)-q\log(1-q)\right) & c<0\\ W_{\max}^{+}\equiv k_{B}T\left(p\log(p/q)-p\log(1-p)\right) & c\geq 0 \end{array}\right.\;, \end{array}$$

which is in both cases independent of the bias for the input process *b*.
